# A Single Dose of Novel PSMA-Targeting Radiopharmaceutical Agent [^177^Lu]Ludotadipep for Patients with Metastatic Castration-Resistant Prostate Cancer: Phase I Clinical Trial

**DOI:** 10.3390/cancers14246225

**Published:** 2022-12-16

**Authors:** Dongho Shin, Seunggyun Ha, Joo Hyun O, Seung ah Rhew, Chang Eil Yoon, Hyeok Jae Kwon, Hyong Woo Moon, Yong Hyun Park, Sonya Youngju Park, Chansoo Park, Dae Yoon Chi, Ie Ryung Yoo, Ji Youl Lee

**Affiliations:** 1 Department of Urology, Seoul St. Mary’s Hospital, College of Medicine, The Catholic University of Korea, Seoul 06591, Republic of Korea; 2 Department of Nuclear Medicine, Seoul St. Mary’s Hospital, College of Medicine, The Catholic University of Korea, Seoul 06591, Republic of Korea; 3 Research Institute of Labeling, FutureChem Co., Ltd., Seoul 04793, Republic of Korea

**Keywords:** metastatic castration-resistant prostate cancer, lutetium-177, PSMA, radiopharmaceutical therapy

## Abstract

**Simple Summary:**

Prostate specific membrane antigen (PSMA) is a transmembrane protein that is highly expressed in prostate cancer cells. For patients with metastatic castration-resistant prostate cancer (mCRPC) who do not respond to conventional treatment, PSMA targeting radiopharmaceutical therapy (RPT) has recently been in the spotlight. [^177^Lu]Ludotadipep is a novel PSMA-targeting therapeutic agent designed with an albumin motif in order to increase the circulation time and uptake in the tumors. The safety and efficacy of [^177^Lu]Ludotadipep were evaluated through a phase I trial.

**Abstract:**

[^177^Lu]Ludotadipep, which enables targeted delivery of beta-particle radiation to prostate tumor cells, had been suggested as a promising therapeutic option for mCRPC. From November 2020 to March 2022, a total of 30 patients were enrolled for single dose of [^177^Lu]Ludotadipep RPT, 6 subjects in each of the 5 different activity groups of 1.9 GBq, 2.8 GBq, 3.7 GBq, 4.6 GBq, and 5.6 GBq. [^177^Lu]Ludotadipep was administered via venous injection, and patients were hospitalized for three days to monitor for any adverse effects. Serum PSA levels were followed up at weeks 1, 2, 3, 4, 6, 8, and 12, and PSMA PET/CT with [^18^F]Florastamin was obtained at baseline and again at weeks 4 and 8. The subjects required positive PSMA PET/CT prior to [^177^Lu]Ludotadipep administration. Among the 29 subjects who received [^177^Lu]Ludotadipep, 36 treatment emergent adverse events (TEAEs) occurred in 17 subjects (58.6%) and 4 adverse drug reactions (ADRs) in 3 subjects (10.3%). Of the total 24 subjects who had full 12-week follow-up data, 16 (66.7%) showed decrease in PSA of any magnitude, and 9 (37.5%) showed a decrease in PSA by 50% or greater. A total of 5 of the 24 patients (20.8%) showed disease progression (PSA increase of 25% or higher from the baseline) at the 12th week following single dose of [^177^Lu]Ludotadipep. These data thus far suggest that [^177^Lu]Ludotadipep could be a promising RPT agent with low toxicity in mCRPC patients who have not been responsive to conventional treatments.

## 1. Introduction

Prostate cancer is the second most common cancer type in males; it is the leading cause of cancer death for men in the United States and the fifth leading cause of death for men in Republic of Korea [1]. Prostate cancer progresses relatively slowly, but biochemical recurrence occurs in about 35% of patients within 10 years after radical prostatectomy [2]. In the initial stage, androgen deprivation therapy is the primary treatment, but after duration of 18 months, only 75% of patients respond to the treatment with the rest converting to castration-resistant prostate cancer (CRPC) [3]. If CRPC is left untreated, the median survival is less than 12 months [4]. Distant metastases have been found in 84% of patients with CRPC, and one third of patients without distant metastases developed bone metastases within 2 years [5].

Prostate specific membrane antigen (PSMA) is a protein that is overexpressed in prostate cancer cells. It has a synergistic correlation with disease progression, and is recognized as a biomarker for diagnosis and treatment of prostate cancer [6]. PSMA protein has enzymatic activity that hydrolyzes N-acetyl-aspartyl-glutamate (NAAG) substrate in vivo to produce N-acetylaspartate and glutamate. A compound chemically modified to prevent degradation of NAAG, the in vivo substrate of PSMA, could become a substance that can specifically target PSMA [7]. Compounds based on glutamate-urea-lysine (GUL) structure are being studied for diagnosis and treatment of prostate cancer [8], and in particular compounds bound with radioisotopes are showing great promise.

Lutetium-177 is a radioactive isotope that emits high energy beta-type particles with the ability to destroy tumor cells. It has a half-life of 6.7 days and a beta energy of 490 keV. The amount of radiation reaching the surrounding normal tissue is relatively small due to the short tissue penetration range of maximum 1.6 mm, and makes Lutetium-177 a suitable radioisotope for therapeutic purposes [9]. Survival gains and patient benefits such as delayed time-to-skeletal-event and pain control were reported following RPT with the most widely studied [^177^Lu]Lutetium-PSMA-617 [10,11], and this radiopharmaceutical was approved by the FDA for patients with mCRPC who progressed after androgen deprivation therapy and taxane based chemotherapy.

Numerous studies have explored various PSMA targeting compounds for diagnostic and therapeutic purposes [12,13]. In order to overcome the short circulation time of the small molecule based [^177^Lu]Lutetium-PSMA-617, a compound was developed with an albumin motif to increase the circulation time and thus the total tumor uptake, [^177^Lu]Ludotadipep, which is also based on the GUL structure. Preclinical treatment effect of this novel radiopharmaceutical on prostate cancer were published earlier [14]. High binding affinity and extended blood circulation time of the compound were observed and showed the potential as an effective RPT. In this prospective, phase I trial, we aimed to investigate the safety and efficacy of [^177^Lu]Ludotadipep, a novel radiopharmaceutical, in patients with mCRPC unresponsive to standard treatment.

## 2. Materials and Methods

### 2.1. [^177^Lu]Ludotadipep

**Figure 1:**
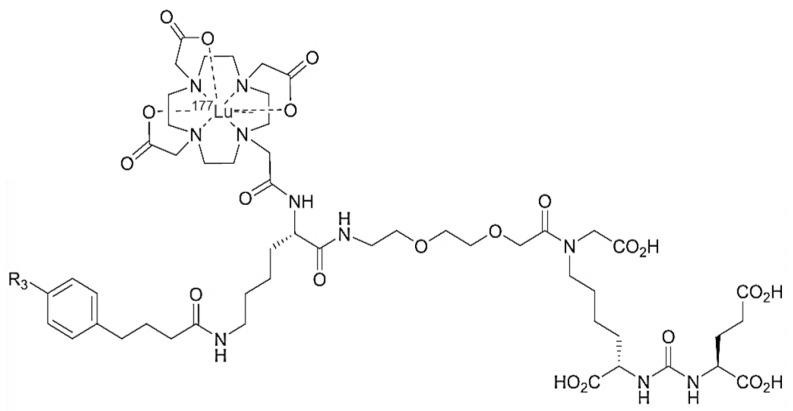
Structure of [^177^Lu]Ludotadipep tested in phase I clinical trial. [^177^Lu]Lutetium (III)2,2′,2′′-(10-((4S,20S,24S)-20,24,26-tricarboxy-15-(carboxymethyl)-4-(4-(4-(4-iodophenyl)butanamido)butyl)-2,5,14,22-tetraoxo-9,12-dioxa-3,6,15,21,23-pentaazahexacosyl)-1,4,7,10-tetraazacyclododecane-1,4,7-triyl)triacetate.

[^177^Lu]Ludotadipep (Figure 1) is a novel PSMA inhibitor labeled with Lu-177 and is characterized by a 4-iodophenyl butanoic group that can bind with albumin, allowing it to stay in the blood for a considerable time and be available longer to be taken up more in prostate cancer cells. [^177^Lu]Ludotadipep was diluted with 0.9% NaCl 5 mL and slowly injected intravenously over 10 min, with 0.9% NaCl slowly administered for 90 min from 30 min before administration of [^177^Lu]Ludotadipep to 60 min after administration. [^177^Lu]Ludotadipep was manufactured and supplied from a GMP site (FutureChem, Seoul, Republic of Korea).

### 2.2. PSMA PET/CT

PSMA PET/CT was performed at screening (baseline), and again at the 4th and 8th weeks after [^177^Lu]Ludotadipep RPT using the diagnostic radiopharmaceutical [^18^F]Florastamin [15]. At 110 min after intravenous injection of 370 ± 37 MBq of [^18^F]Florastamin (FutureChem, Seoul, Korea), PET/CT images were acquired. A focal uptake higher than the background level that was not associated with physiologic uptake or known pitfall was defined as positive.

### 2.3. Study Method

This was a prospective, single center, phase I open label study testing single administration of a radiopharmaceutical. Patients were required to have an Eastern Cooperative Oncology Group (ECOG) performance status score of 2 or lower. Subjects were screened with PSMA PET/CT for lesions showing PSMA-RADS 4 or 5 [16]. Five groups of up to six subjects were administered once with [^177^Lu]Ludotadipep at doses of 1.9 GBq, 2.8 GBq, 3.7 GBq, 4.6 GBq, and 5.6 GBq. The dose was increased sequentially if two or less out of six subjects were confirmed with dose limiting toxicity (DLT). The subjects were hospitalized for three days following the administration of [^177^Lu]Ludotadipep to monitor for any immediate adverse effect.

Serum prostate specific antigen (PSA) levels were tested 1, 2, 3, 4, 6, 8, and 12 weeks after administration of [^177^Lu]Ludotadipep and compared with the baseline PSA measurements. We reviewed the occurrence of adverse events at the relevant time points in the outpatient clinics. At four and eight weeks after administration, the PSMA PET/CT was repeated for qualitative and quantitative comparison of the metastatic lesions.

### 2.4. Outcomes

The primary outcome was the DLT, defined as on common terminology criteria for adverse events (CTCAE) v5.0: grade 4 thrombocytopenia (platelet level < 25 × 10^9^/L), grade 4 neutropenia (absolute neutrophil count, ANC < 0.5 × 10^9^/L), grade 3 febrile neutropenia (ANC < 1.0 × 10^9^/L with a body temperature exceeding 38.3 °C on at least 1 occasion or a body temperature above 38.0 °C persisting for 1 h), or grade 3 to 4 non-hematological toxicity resulting from [^177^Lu]Ludotadipep that lasts more than five days. The maximum tolerated dose (MTD) for phase II was to be determined based on incidence of the DLT.

The secondary outcomes included (1) safety evaluation based on symptoms, physical examinations, and laboratory tests; (2) PSA level assessment; and (3) imaging assessment based on PSMA PET/CT.

### 2.5. Statistical Analysis

Statistical analyses were performed using SAS software version 9.4 (SAS institute, Cary, NC, USA). Demographic information, safety assessment, efficacy analyses based on PSA responses, and PET/CT findings are described from all subjects who received [^177^Lu]Ludotadipep. DLT assessment was carried out in those who completed the follow up scheme. Median ± standard deviation (SD) are described for continuous parameters.

### 2.6. Ethical Statement

This study was approved by Seoul St. Mary’s Hospital Institutional Review Board (IRB no. KC20MDSF0483) and the Korean Ministry of Food and Drug Safety (KMFDS). Written informed consent was obtained from all subjects, and the study was in compliance with the Helsinki Declaration and local regulations. (ClinicalTrials.gov Identifier: NCT04509557).

## 3. Results

### 3.1. Patient Characteristics

From November 2020 to March 2022, 42 men with mCRPC in whom disease had progressed after standard treatments were recruited for screening, and 12 men were excluded since they did not meet the inclusion/exclusion criteria (n = 6), withdrew consent (n = 2), or for other causes (n = 4; such as COVID19 quarantine). 30 patients were enrolled and 29 received [^177^Lu]Ludotadipep (1 subject found to deviate from the inclusion/exclusion criteria after enrollment). A total of 24 subjects completed the follow-up protocol, while 5 patients withdrew after administration of [^177^Lu]Ludotadipep (1 withdrawal of consent; 4 at the discretion of the investigator for other treatments including one subject admitted for treatment of COVID19 infection). Participants for the higher [^177^Lu]Ludotadipep dose group were recruited after the DLT was confirmed from two or less of six subjects in a given dose group.

Mean age was 72.7 ± 8.1 years, and the mean PSA level prior to treatment was 681.3 ± 1139.9 ng/mL. The baseline characteristics of the 29 subjects who received the investigational RPT are shown in Table 1.

### 3.2. Treatment Related Toxicity

Adverse events attributed to [^177^Lu]Ludotadipep are shown in Table 2.

Total 24 subjects had full DLT assessment (4 from 1.9 GBq, 6 from 2.8 GBq, 6 from 3.7 GBq, 5 from 4.6 GBq, and 3 from 5.6 GBq group). No DLT occurred at any level of the 5 tested dose groups.

The treatment emergent adverse events (TEAEs) according to system organ class (SOC) and preferred term (PT) are also shown in Table 3. Among the 29 subjects, 36 TEAEs occurred in 17 subjects (58.6%) including 5 events that occurred in 4 subjects (66.7%) in the 1.9 GBq group, 3 events that occurred in 2 subjects (33.3%) in the 2.8 GBq group, 6 events that occurred in 3 subjects (50.0%) in the 3.7 GBq group, 9 events that occurred in 4 subjects (66.7%) in the 4.6 GBq group, and 13 events that occurred in 4 subjects (80.0%) in the 5.6 GBq group. In terms of severity, most of the TEAEs were grade 1 (29/36 events) or grade 2 (5/36 events). Two out of 36 TEAEs were grade 3, and they were not serious adverse events (SAE) and not related to the [^177^Lu]Ludotadipep administration. A single SAE was reported by one subject in the [^177^Lu]Ludotadipep 5.6 GBq group (asthenia), but it was considered not related to the [^177^Lu]Ludotadipep administration and the severity was grade 1.

Adverse drug reactions (ADRs) according to SOC and PT are shown in Table 4. A total four ADRs occurred in three subjects, including one event that occurred in one subject each in the 1.9 GBq and 3.7 GBq groups and two events that occurred in one subject in the 2.8 GBq group. No ADR occurred in the [^177^Lu]Ludotadipep 4.6 GBq and 5.6 GBq groups. In terms of severity, most of the ADRs were grade 1 (3/4 events) or grade 2 (1/4 events), and all were recovered (one recovering). No serious ADR was reported in any of the dose groups.

There were no adverse event or ADR leading to withdrawal from the study or death in any of the dose groups. Overall summary of TEAEs and ADRs are shown in Supplementary Tables S1 and S2.

### 3.3. Laboratory Changes

There were no significant changes in the serum hemoglobin (Hb), leukocyte, platelet, absolute neutrophil counts (ANC), and sodium levels (Supplementary Table S3) before and after the administration of [^177^Lu]Ludotadipep.

### 3.4. PSA Response

The detailed PSA levels at weeks 1, 2, 3, 4, 6, 8, and 12 after [^177^Lu]Ludotadipep administration are shown in Supplementary Table S4. At 12 weeks, the 1.9 GBq and 2.8 GBq groups showed increases of 15.15 and 5.00 ng/mL in median absolute PSA levels, respectively, compared to baseline. In the 3.7 GBq and higher dose groups, decrease in median PSA level was observed compared to the baseline values. Among the dose groups, the 5.6 GBq treated group had the greatest drop in the median absolute PSA level of 87.00 ng/mL from baseline to 12 weeks.

**Figure 2:**
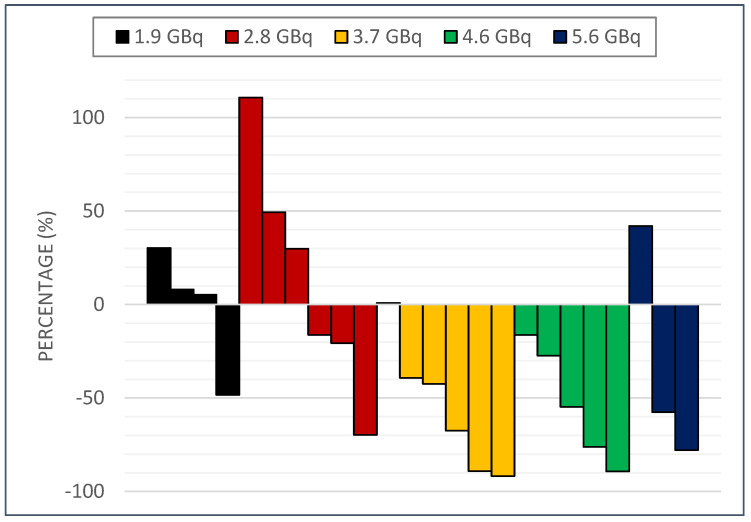
Waterfall plots by administered dose showing percentage change of best PSA response per subject.

Figure 2 shows waterfall plot for percentage change of each subject’s best PSA response. Supplementary Figure S1 shows spider plots for PSA levels from baseline to 1, 2, 3, 4, 6, 8, and 12 weeks after [^177^Lu]Ludotadipep administration.

Of the total 24 patients with full set of follow-up data for 12 weeks, 16 (66.7%) showed any decrease in PSA, 12 (50%) showed a decrease of PSA by more than 30%, and 9 (37.5%) showed a decrease in PSA by more than 50%. Overall, 5 out of 24 patients (20.8%) showed disease progression (25% or greater increase in PSA from the baseline) at the end of the study at the 12th week following [^177^Lu]Ludotadipep administration.

### 3.5. Radiological Assessment

Three sets of PSMA PET/CT images, at baseline, 4 weeks and 8 weeks after [^177^Lu]Ludotadipep administration were available in a total of 25 subjects. Mean (SD) of peak standardized uptake values corrected for lean body mass (SUL_peak_) measured from PSMA PET/CT at baseline were 16.2 (12.0), 24.4 (22.0), 10.3 (5.9), 17.5 (11.9), and 20.1 (18.1) in the [^177^Lu]Ludotadipep 1.9 GBq, 2.8 GBq, 3.7 GBq, 4.6 GBq, and 5.6 GBq groups, respectively. The mean SUL_peak_ in the Week 4 and Week 8 PET/CT images decreased compared to baseline in all dose groups (Supplementary Figure S2). However, in the [^177^Lu]Ludotadipep 1.9 GBq and 5.6 GBq groups, the week 8 PSMA PET/CT SUL_peak_ drops were not greater than the week 4 drops.

**Figure 3:**
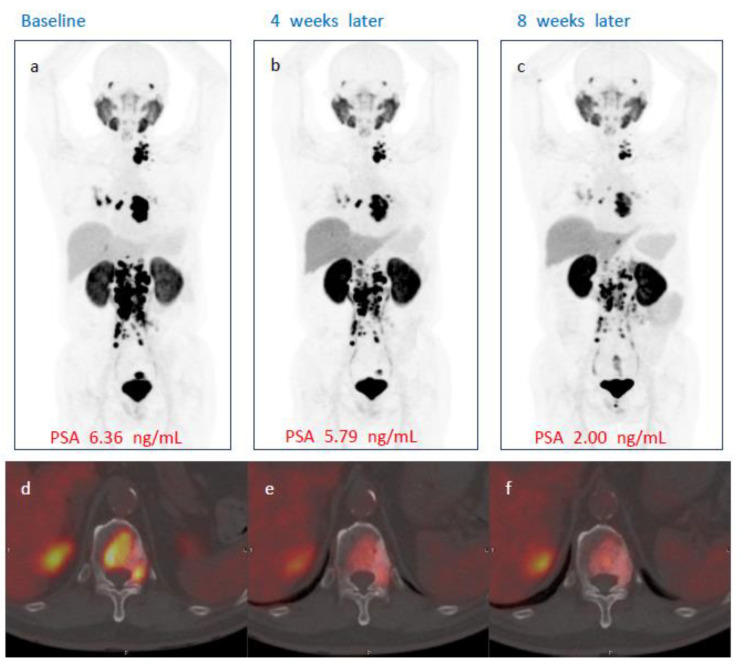
A case of a subject showing PSA response to [^177^Lu]Ludotadipep. (**a**,**d**) Baseline [^18^F]Florastamin PSMA PET/CT maximum intensity projection (MIP) and fused axial images with multiple bone and lymph node metastases. (**b**,**c**) PSMA PET/CT MIP images at four and eight weeks after administration of [^177^Lu]Ludotadipep showing decreased tumor burden. (**e**,**f**) Axial fused PSMA PET/CT images at four and eight weeks show decreased uptake in metastatic lesion in the thoracic vertebra. The patient’s PSA decreased from 6.36 to 2.00 ng/mL at 8 weeks.

**Figure 4:**
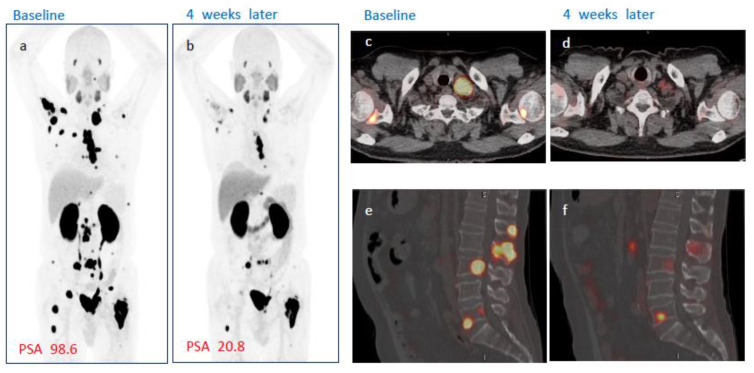
A case of a subject showing response to [^177^Lu]Ludotadipep**.** (**a**,**c**,**e**) Baseline [^18^F]Florastamin PSMA PET/CT MIP, fused axial and sagittal images. (**b**,**d**,**e**) PSMA PET/CT images four weeks after administration of [^177^Lu]Ludotadipeps (**a**,**b**) MIP images show decreased tumor burden in the bone lesions and lymph nodes. (**c**,**d**) Axial fused PSMA PET/CT images show marked regression of left supraclavicular lymph node and bone metastases. (**e**,**f**) Sagittal PSMA PET/CT images show decreased uptake in the spine lesions.

No patient demonstrated complete response on either week 4 or week 8 PSMA PET/CT after single administration of [^177^Lu]Ludotadipep. However, on week 8 PSMA PET/CT, 14 (56%) subjects had partial response and 10 subjects (40%) had stable disease (SD). One subject demonstrated progression on imaging. The overall objective response rate (ORR) was 40% at week 4 and 56% at week 8. The overall disease control rate (DCR) was 92% at week 4 and 96% at week 8 according to PSMA PET/CT findings. A case of a subject who received 3.7 GBq of [^177^Lu]Ludotadipep is shown in Figure 3, and another case of a subject who received 4.6 GBq is shown in Figure 4.

## 4. Discussion

[^177^Lu]Ludotadipep is a novel radiopharmaceutical based on the chemical structure of GUL (glutamate-urea-lysine), and is composed of relatively hydrophilic residues. It has a 4-iodophenyl butanoic group that interacts with serum albumin to elongate the circulation time of the compound and subsequently increase the uptake of the therapeutic radiopharmaceutical by prostate cancer cells.

Following single cycle of [^177^Lu]Ludotadipep, PSA drop of 50% or greater was seen in 9 out of 24 subjects with full 12 weeks of follow-up data. In the TheraP trial, 66% of the patients showed PSA response after up to 6 cycles of [^177^Lu]Lu-PSMA-617 RPT [10]. In the VISION Trial, the patients received 4 to 6 cycles of [^177^Lu]Lu-PSMA-617 and PSA decrease of 50% or greater was seen in 46.0%, compared to the rate of 7.1% in the standard care alone group [11]. In this study, any decrease in PSA was noted in 12 of 14 (85.7%) subjects in the 3.7 to 5.6 GBq dose groups, and the overall radiological disease control rate was 96% on week 8 PSMA PET/CT. In this phase I study, the administered radiopharmaceutical activity was lower than the TheraP and VISION trials (doses of 6.0 to 8.5 GBq, and 7.4 GBq, respectively), and only single cycle rather than repeated cycles was tested. At all visits, the proportion of subjects showing PSA decrease was higher in the [^177^Lu]Ludotadipep 3.7 to 5.6 GBq groups than in the [^177^Lu]Ludotadipep 1.9 GBq and 2.8 GBq groups. Our results suggest favorable control rates compared to other early phase trials of novel PSMA targeting radiopharmaceuticals [17,18]. Though direct comparison with [^177^Lu]Lu-PSMA-617 or other radiopharmaceuticals is not possible at this early stage, favorable PSA and PSMA PET/CT responses support further trials with repeated cycles of higher doses of [^177^Lu]Ludotadipep.

As with [^177^Lu]Lu-PSMA-617 [19], [^177^Lu]Ludotadipep showed mostly renal clearance. Detailed biodistribution and dosimetry analyses for [^177^Lu]Ludotadipep are ongoing for publication, and show kidneys and bone marrow to be the critical organs for [^177^Lu]Ludotadipep. The serum creatinine levels did not show difference after administration, and no TEAEs or ADRs related to kidney injury occurred in this study. Grade 3 to 5 renal effects were comparable in the [^177^Lu]Lu-PSMA-617 plus standard care group and standard care alone group (3.4% and 2.9%) in the VISION trial [11]. Though the incidence may not be high, nephrotoxicity was also observed after treatment with [^177^Lu]Lu-Octreotate [20], and kidney damage should be monitored and investigated in upcoming clinical trials. Marrow suppression was observed with [^177^Lu]Lu-PSMA-617 [11], but it could also be a manifestation in patients with extensive bone metastases in advanced prostate cancer. Longer follow-up of TheraP population did not raise additional safety issues [21]. Though severe marrow suppression did not develop during the 12 weeks of testing in this study, possibly due to the lower administered activity of the radiopharmaceutical, caution would be mandatory following higher activity RPT and with patients demonstrating high tumor burden in the skeletal lesions.

As could be expected from the high salivary gland uptake in the PSMA PET/CT images, xerostomia was the most frequent non-hematologic side effect in this study, though serum amylase level was not checked for the diagnosis. Previous studies have shown high uptake of the [^177^Lu]Lu-PSMA-based radiopharmaceuticals by the salivary glands [8,22]. A recent study showed that mild xerostomia occurred in 2 out of 56 patients after 3 to 4 cycles of treatment but spontaneously resolved before 3 months [23] and was recoverable [24], and applying external cooling with ice packs may reduce the salivary gland uptakes [25]. It is necessary to learn how to further reduce xerostomia for futureapplications.

The lack of data on long-term safety, survival outcomes, and changes in patient reported bone pain or performance are among the limitations of this study, and will be addressed in future trials. In this pilot study of single cycle of [^177^Lu]Ludotadipep, antitumor effects were observed in majority of the subjects. Phase II clinical trial with repeated cycles are ongoing, and the safety profile and therapeutic effect will be further assessed.

## 5. Conclusions

[^177^Lu]Ludotadipep led to PSA drop of 50% or greater in 37.5% of subjects with mCRPC after single administration, and showed no serious treatment-related side effects. [^177^Lu]Ludotadipep has the potential to be an effective option for mCRPC patients who have not responded to previous treatments, and phase II study of multi-cycle [^177^Lu]Ludotadipep RPT is ongoing.

## Figures and Tables

**Table 1:** Baseline characteristics.

Characteristics	N = 29
Age (years)	72.7 ± 8.1
Time from prostate cancer diagnosis (months)	67.0 ± 50.8
**ECOG Performance Status**	
0	26 (89.6%)
1	3 (10.34%)
PSA (ng/mL)	681.3 ± 1139.9
**Gleason Score**	
3 + 4	2 (6.9%)
4 + 3	2 (6.9%)
4 + 4	9 (31.0%)
4 + 5	9 (31.0%)
5 + 4	5 (17.2%)
5 + 5	2 (6.9%)
Bone metastasis	28 (96.6%)
Lymph node metastasis	18 (62.1%)
Liver metastasis	4 (13.8%)
Lung metastasis	5 (17.2%)
Radical prostatectomy	16 (55.2%)
Radiation Therapy	7 (24.1%)
**Prior Systemic Treatment**	
Androgen deprivation therapy only	7 (24.1%)
Docetaxel	3 (10.3%)
Abiraterone	7 (24.1%)
Enzalutamide	1 (3.4%)
Docetaxel then enzalutamide	4 (13.8%)
Docetaxel then abiraterone	3 (10.3%)
Degarelix then androgen deprivation therapy	1 (3.4%)
Abiraterone then docetaxel then enzalutamide	3 (10.3%)

**Table 2:** Adverse events (AEs) depending on doses.

AEs/Dose	1.9 GBq	2.8 GBq	3.7 GBq	4.6 GBq	5.6 GBq
	(N = 6)	(N = 6)	(N = 6)	(N = 6)	(N = 5)
**Hematologic**					
Neutropenia	0	0	0	0	0
Febrile neutropenia	0	0	0	0	0
Anemia	1 (16.7%)	0	1 (16.7%)	0	1 (20.0%)
Thrombocytopenia	0	0	0	0	0
**Non-hematologic**					
Anorexia	0	1 (16.7%)	1 (16.7%)	0	1 (20.0%)
Dyspnea	0	0	0	0	0
Fatigue	0	0	0	0	0
Nausea	1 (16.7%)	1 (16.7%)	1 (16.7%)	0	2 (40.0%)
Stomatitis	0	0	0	0	0
Vomiting	0	0	0	0	0
Weight loss	0	0	0	0	0
Constipation	0	0	0	1 (16.7%)	1 (16.7%)
Xerostomia	1 (16.7%)	1 (16.7%)	1 (16.7%)	1 (16.7%)	2 (20.0%)

**Table 3:** Treatment emergent adverse events (TEAEs) according to system organ class (SOC) and preferred term (PT).

	Total 29 Subjects
**Subjects with TEAEs, n(%) [number of events]**	**17(58.6) [36]**
**Gastrointestinal disorders**	**6(20.7) [8]**
Nausea	5(17.2) [5]
Constipation	2(6.9) [2]
Hematochezia	1(3.5) [1]
**Blood and lymphatic system disorders**	**3(10.3) [3]**
Anemia	3(10.3) [3]
**Laboratory investigations**	**3(10.3) [3]**
Alanine aminotransferase increased	1(3.5) [1]
Aspartate aminotransferase increased	1(3.5) [1]
Platelet count decreased	1(3.5) [1]
**Metabolism and nutrition disorders**	**3(10.3) [3]**
Decreased appetite	3(10.3) [3]
**Nervous system disorders**	**3(10.3) [3]**
Headache	2(6.9) [2]
Dizziness	1(3.5) [1]
**General disorders and administration site conditions**	**2(6.9) [3]**
Edema peripheral	1(3.5) [2]
Asthenia	1(3.5) [1]
**Psychiatric disorders**	**1(3.5) [2]**
Insomnia	1(3.5) [2]
**Injury, poisoning and procedural complications**	**1(3.5) [1]**
Procedural pain	1(3.5) [1]
**Musculoskeletal and connective tissue disorders**	**2(6.9) [1]**
Bone pain	1(3.5) [1]
Arthralgia	1(3.5) [1]
**Renal and urinary disorders**	**1(3.5) [1]**
Dysuria	1(3.5) [1]
**Reproductive system and breast disorders**	**1(3.5) [1]**
Scrotal pain	1(3.5) [1]
**Dry mouth**	**6(20.7) [6]**
**Skin and subcutaneous tissue disorders**	**1(3.5) [1]**
Dermatitis	1(3.5) [1]

**Table 4:** Adverse drug reactions (ADRs) according to SOC and PT.

	Total 29 Subjects
**Subjects with ADRs, n(%) [number of events]**	**3(10.3)** [4]
**Gastrointestinal disorders**	
Nausea	2(6.9) [2]
**Metabolism and nutrition disorders**	
Decreased appetite	1(3.5) [1]
**Skin and subcutaneous tissue disorders**	
Dermatitis	1(3.5) [1]

## Data Availability

The data presented in this study are available upon reasonable request.

## Supplementary Materials

The following supporting information can be downloaded at: https://www.mdpi.com/article/10.3390/cancers14246225/s1, Supplementary Table S1: Overall summary of TEAEs; Supplementary Table S2: Overall summary of ADRs; Supplementary Table S3: Change in hemoglobin, leukocyte, platelet, ANC; Supplementary Table S4. The PSA levels at Week 1, 2, 3, 4, 6, 8, and 12 after [^177^Lu]Ludotadipep administration; Supplementary Figure S1: Spider plots for PSA level Week 1, 2, 3, 4, 6, 8, 12 after [^177^Lu]Ludotadipep administration; Supplementary Figure S2: Waterfall plots of percent changes in SUL_peak_ on PSMA PET/CT (a) at Week 4 after [^177^Lu]Ludotadipep administration, and (b) at Week 8.
