# Supplementary material for: A Single Dose of Novel PSMA-Targeting Radiopharmaceutical Agent [177Lu]Ludotadipep for Patients with Metastatic Castration-Resistant Prostate Cancer: Phase I Clinical Trial
Source: Cancers (Basel). 2022 Dec 16;14(24):6225. doi: 10.3390/cancers14246225 (PMC9777064; doi:10.3390/cancers14246225)

Supplementary Table S1. Overall summary of TEAEs

Items		Ludotadipep 1.9 GBq (N=6)	Ludotadipep 2.8 GBq (N=6)	Ludotadipep 3.7 GBq (N=6)	Ludotadipep 4.6 GBq (N=6)	Ludotadipep 5.6 GBq (N=5)	Total (N=29)
Subjects with TEAEs, n(%) [event]		4(66.7) [5]	2(33.3) [3]	3(50.0) [6]	4(66.7) [9]	4(80.0) [13]	17(58.6) [36]
Severity	Grade 1	1	2	5	9	12	29
	Grade 2	3	1	1	0	0	5
	Grade 3	1	0	0	0	1	2
	Grade 4	0	0	0	0	0	0
	Grade 5	0	0	0	0	0	0
Relation	Obvious	0	0	0	0	0	0
	Probably related	0	1	0	2	2	5
	Possibly related	1	0	0	0	0	1
	Barely possible	0	2	1	0	0	3
	Not related	4	0	5	7	11	27
	Not assessable	0	0	0	0	0	0
Outcome	Recovered	4	3	2	7	10	26
	Recovering	0	0	4	2	2	8
	Not recovered	1	0	0	0	1	2
	Recovered with sequelae	0	0	0	0	0	0
	Death	0	0	0	0	0	0
	Unknown	0	0	0	0	0	0
Action taken [a)]	None	1	0	1	0	1	3
	Concomitant	2	3	3	9	8	25
	Hospitalization or extension of hospitalization	0	0	0	0	1	1
	Curative or diagnostic procedure	2	0	1	0	1	4
	Other	1	0	2	0	2	5
Subjects with Serious AEs, n(%) [event]		0(0.0) [0]	0(0.0) [0]	0(0.0) [0]	0(0.0) [0]	1(20.0) [1]	1(3.5) [1]
Subjects with AEs Leding to Drug withdrwan, n(%) [event]		0(0.0) [0]	0(0.0) [0]	0(0.0) [0]	0(0.0) [0]	0(0.0) [0]	0(0.0) [0]
Subjects with AEs Leading to Death, n(%) [event]		0(0.0) [0]	0(0.0) [0]	0(0.0) [0]	0(0.0) [0]	0(0.0) [0]	0(0.0) [0]

a) Action taken can be duplicated.

Supplementary Table S2. Overall summary of ADRs

Items		Ludotadipep 1.9 GBq (N=6)	Ludotadipep 2.8 GBq (N=6)	Ludotadipep 3.7 GBq (N=6)	Ludotadipep 4.6 GBq (N=6)	Ludotadipep 5.6 GBq (N=5)	Total (N=29)
Subjects with ADRs, n(%) [event]		1(16.7) [1]	1(16.7) [2]	1(16.7) [1]	0(0.0) [0]	0(0.0) [0]	3(10.3) [4]
Severity	Grade 1	1	1	1	0	0	3
	Grade 2	0	1	0	0	0	1
	Grade 3	0	0	0	0	0	0
	Grade 4	0	0	0	0	0	0
	Grade 5	0	0	0	0	0	0
Outcome	Recovered	1	2	0	0	0	3
	Recovering	0	0	1	0	0	1
	Not recovered	0	0	0	0	0	0
	Recovered with sequelae	0	0	0	0	0	0
	Death	0	0	0	0	0	0
	Unknown	0	0	0	0	0	0
Action taken [a)]	None	1	0	0	0	0	1
	Concomitant	0	2	1	0	0	3
	Hospitalization or extension of hospitalization	0	0	0	0	0	0
	Curative or diagnostic procedure	0	0	0	0	0	0
	Other	0	0	0	0	0	0
Subjects with Serious ADRs, n(%) [event]		0(0.0) [0]	0(0.0) [0]	0(0.0) [0]	0(0.0) [0]	0(0.0) [0]	0(0.0) [0]
Subjects with ADRs Leding to Drug withdrwan, n(%) [event]		0(0.0) [0]	0(0.0) [0]	0(0.0) [0]	0(0.0) [0]	0(0.0) [0]	0(0.0) [0]
Subjects with ADRs Leading to Death, n(%) [event]		0(0.0) [0]	0(0.0) [0]	0(0.0) [0]	0(0.0) [0]	0(0.0) [0]	0(0.0) [0]

a) Action taken can be duplicated.

Supplementary Table S3. Change in hemoglobin, leukocyte, platelet, ANC and sodium levels

Serum	Initial	12wk
Hemoglobin (g/dl)	12.0 ± 1.3	11.7 ± 1.5
Leukocyte (10^9/L)	7.0 ± 2.1	6.6 ± 2.5
Platelet (10^9/L)	247.8 ± 120.6	218.8 ± 100.2
Absolute neutrophil count (10^9/L)	4.4 ± 1.5	5.1 ± 4.1
Creatinine (mg/dl)	0.9 ± 0.3	0.9 ± 0.3
Sodium (mmol/L)	141 ± 8.3	140 ± 7.6

Supplementary Table S4. The PSA levels at Week 1, 2, 3, 4, 6, 8, 12 after [^{177}Lu]Ludotadipep administration**.**

Statistics	Ludotadipep 1.9 GBq (N=6)	Ludotadipep 2.8 GBq (N=6)	Ludotadipep 3.7 GBq (N=6)	Ludotadipep 4.6 GBq (N=6)	Ludotadipep 5.6 GBq (N=5)	Total (N=29)
**Screening**						
Number	6	6	6	6	5	29
Mean (SD)	467.58(999.0)	185.7(201.2)	67.3(59.2)	1074.3(1180.9)	1339.0(1126.5)	602.2 (929.2)
Median	75.7	119.0	60.2	786.5	1090.0	123.0
Min : Max	7.86 : 2504.0	18.7 : 545.0	5.3 : 149.0	58.7 : 2856.0	230.0 : 2986.0	5.3 : 2986.0
**Week 1**						
Number	4	5	5	6	4	24
Mean (SD)	92.7(99.0)	214.6(181.8)	87.8(72.7)	1544.1(1686.3)	1791.3(2220.8)	763.0 (1362.0)
Median	71.0	236.0	74.8	1034.0	975.0	162.5
Min : Max	8.8 : 220.0	24.3 : 432.0	8.9 : 174.0	76.1 : 3637.0	215.0 : 5000.0	8.8 : 5000.0
**Week 2**						
Number	4	6	6	5	3	24
Mean (SD)	103.5(111.7)	249.7(218.8)	69.4(75.1)	1521.3(1981.9)	836.7(872.2)	518.5 (1046.8)
Median	81.8	231.2	45.9	133.0	520.0	122.5
Min : Max	8.3 : 242.0	25.2 : 488.0	6.1 : 197.0	55.3 : 4012.0	167.0 : 1823.0	6.1 : 4012.0
**Week 3**						
Number	3	6	6	5	3	23
Mean (SD)	140.0(135.1)	290.6(282.7)	64.9(77.7)	1318.2(1718.0)	758.0(653.2)	496.5 (916.0)
Median	132.0	210.3	44.9	117.0	712.0	117.0
Min : Max	9.1 : 279.0	25.7 : 636.0	4.9 : 207.0	32.9 : 3414.0	129.0 : 1433.0	4.9 : 3414.0
**Week 4**						
Number	5	6	6	5	3	25
Mean (SD)	100.5(115.6)	280.5(270.9)	50.8(57.4)	1102.2(1429.7)	677.7(589.3)	401.4 (743.6)
Median	45.1	202.5	34.3	106.0	642.0	76.5
Min : Max	11.0 : 285.0	26.9 : 635.0	3.9 : 150.0	20.8 : 2717.0	107.0 : 1284.0	3.9 : 2717.0
**Week 6**						

Statistics	Ludotadipep 1.9 GBq (N=6)	Ludotadipep 2.8 GBq (N=6)	Ludotadipep 3.7 GBq (N=6)	Ludotadipep 4.6 GBq (N=6)	Ludotadipep 5.6 GBq (N=5)	Total (N=29)
Number	5	6	6	5	3	25
Mean (SD)	101.8(109.7)	286.9(306.5)	35.5(48.1)	795.3(1106.0)	684.3(875.3)	338.9 (619.3)
Median	40.9	184.3	19.9	96.5	319.0	65.5
Min : Max	12.3 : 259.0	26.8 : 795.0	2.6 : 128.0	8.6 : 2536.0	50.8 : 1683.0	2.6 : 2536.0
**Week 8**						
Number	5	6	6	5	3	25
Mean (SD)	88.2(83.2)	290.5(317.5)	34.8(47.3)	589.7(906.3)	986.2(1468.5)	332.0 (664.4)
Median	43.8	148.7	14.9	120.0	208.0	70.6
Min : Max	17.0 : 184.0	29.3 : 746.0	2.0 : 124.0	6.8 : 2158.0	70.6 : 2680.0	2.0 : 2680.0
**Week 12**						
Number	4	6	6	5	3	24
Mean (SD)	58.1(54.2)	226.72(222.7)	34.7(51.3)	555.5(867.30	1785.0(2784.5)	413.9 (1065.4)
Median	44.3	151.8	11.6	184.0	212.0	54.2
Min : Max	8.9 : 135.0	33.7 : 540.0	3.5 : 136.0	12.9 : 2076.0	143.0 : 5000.0	3.5 : 5000.0
**Change from Screening at Week 1 [a)]**						
Number	4	5	5	6	4	24
Mean (SD)	25.2(46.6)	17.0(86.6)	8.1(31.3)	469.8(667.1)	595.5(977.3)	226.1 (537.7)
Median	2.4	12.2	2.7	247.5	227.0	18.6
Min : Max	1.0 : 95.0	-113.0 : 124.0	-35.2 : 51.0	17.4 : 1742.0	-86.0 : 2014.0	-113.0 : 2014.0
**Change from Screening at Week 2 [a)]**						
Number	4	6	6	5	3	24
Mean (SD)	35.9(55.4)	63.95(155.4)	2.1(42.9)	523.9(914.5)	237.7(432.2)	161.37 (457.0)
Median	13.2	12.7	2.2	19.0	43.0	11.8
Min : Max	0.3 : 117.0	-57.00 : 367.0	-45.7 : 74.0	-7.7 : 2117.0	-63.0 : 733.0	-63.0 : 2117.0
**Change from Screening at Week 3 [a)]**						
Number	3	6	6	5	3	23
Mean (SD)	55.8(85.3)	104.8(198.3)	-2.37(54.4)	320.9(672.0)	159.0(231.6)	124.5 (333.7)

Statistics	Ludotadipep 1.6 GBq (N=6)	Ludotadipep 2.8 GBq (N=6)	Ludotadipep 3.7 GBq (N=6)	Ludotadipep 4.6 GBq (N=6)	Ludotadipep 5.6 GBq (N=5)	Total (N=29)
Median	12.0	46.3	0.3	3.0	235.0	12.0
Min : Max	1.3 : 154.0	-57.5 : 496.0	-68.1 : 84.0	-30.1 : 1519.0	-101.0 : 343.0	-101.0 : 1519.0
**Change from Screening at Week 4 [a)]**						
Number	5	6	6	5	3	25
Mean (SD)	40.2(67.5)	94.7(179.1)	-16.5(38.5)	104.9(349.7)	78.7(175.3)	57.2 (181.6)
Median	13.7	46.2	-0.9	-9.5	165.0	3.4
Min : Max	2.2 : 160.0	-66.2 : 444.0	-72.5 : 27.0	-139.0 : 723.0	-123.0 : 194.0	-139.0 : 723.0
**Change from Screening at Week 6 [a)]**						
Number	5	6	6	5	3	25
Mean (SD)	41.6(55.9)	101.2(170.8)	-31.8(44.5)	-202.1(270.8)	85.3(439.8)	-5.2 (218.9)
Median	9.5	27.9	-12.6	-54.4	-158.0	4.4
Min : Max	4.4 : 134.0	-76.9 : 370.0	-109.3 : 5.0	-626.0 : 7.5	-179.2 : 593.0	-626.0 : 593.0
**Change from Screening at Week 8 [a)]**						
Number	5	6	6	5	3	25
Mean (SD)	27.9(25.9)	104.7(222.0)	-32.5(50.6)	-407.6(585.3)	387.2(1043.1)	-12.2 (463.8)
Median	12.4	21.2	-4.9	-56.2	-159.4	-1.8
Min : Max	5.8 : 59.0	-80.0 : 512.0	-124.5 : 1.0	-1306.0 : 61.3	-269.0 : 1590.0	-1306.0 : 1590.0
**Change from Screening at Week 12 [a)]**						
Number	4	6	6	5	3	24
Mean (SD)	12.8(16.1)	41.0(149.3)	-32.6(54.9)	-441.9(658.4)	1186.0(2360.7)	60.4 (887.9)
Median	15.2	5.0	-9.3	-62.5	-87.0	-6.7
Min : Max	-8.3 : 29.0	-88.0 : 333.0	-132.7 : 13.0	-1442.0 : 125.3	-265.0 : 3910.0	-1442.0 : 3910.0

a) Change = PSA level at visit 4, 5, 6, 7, 8, 9, 10 – PSA level at screening

Supplementary Figure S1. Spider plots for PSA level Week 1, 2, 3, 4, 6, 8, 12 after [^{177}Lu]Ludotadipep administration

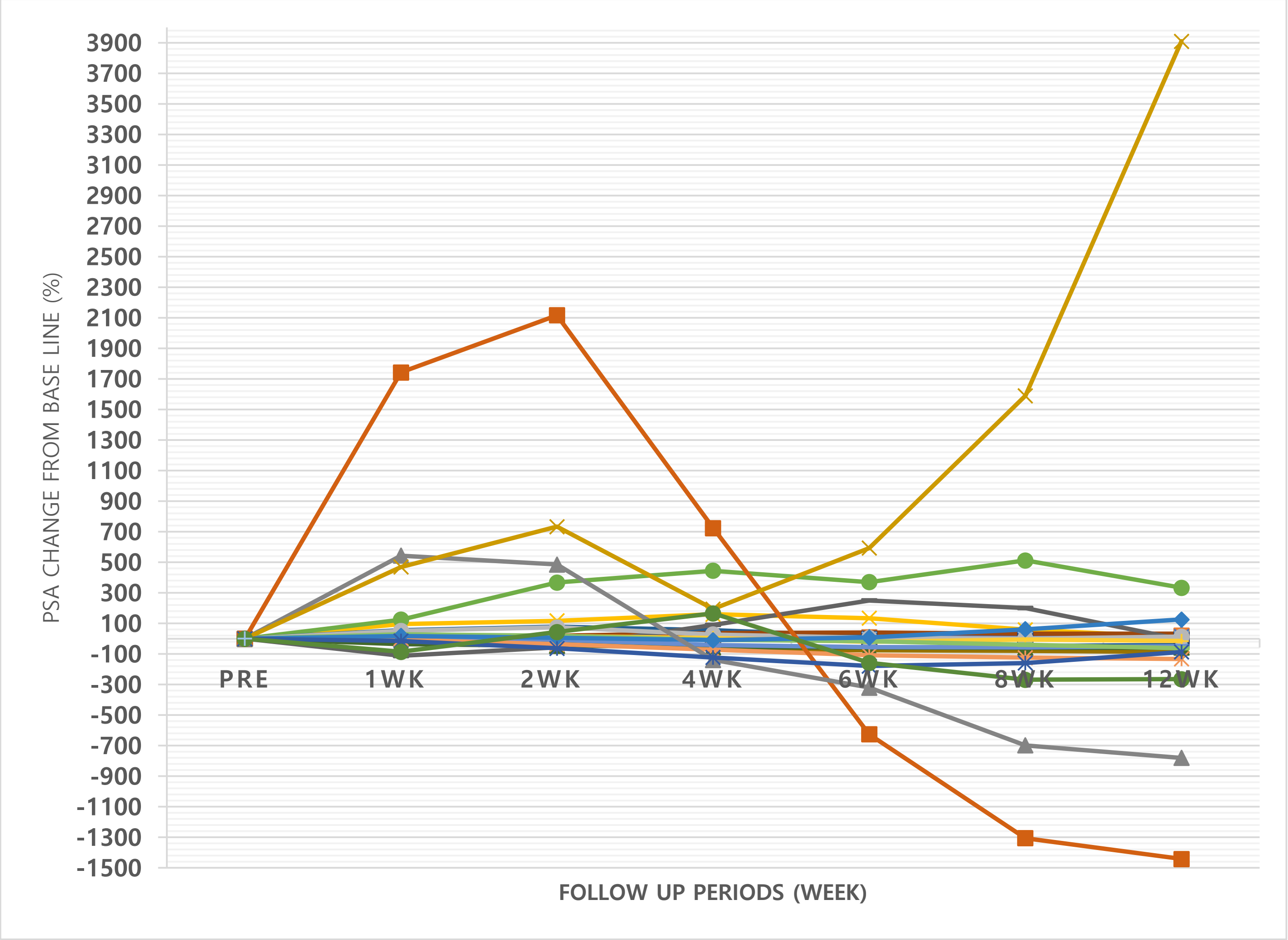

Supplementary Figure S2. Waterfall plots of percent changes in SULpeak on PSMA PET/CT (a) at week 4 after [^{177}Lu]Ludotadipep administration, and (b) at week 8.

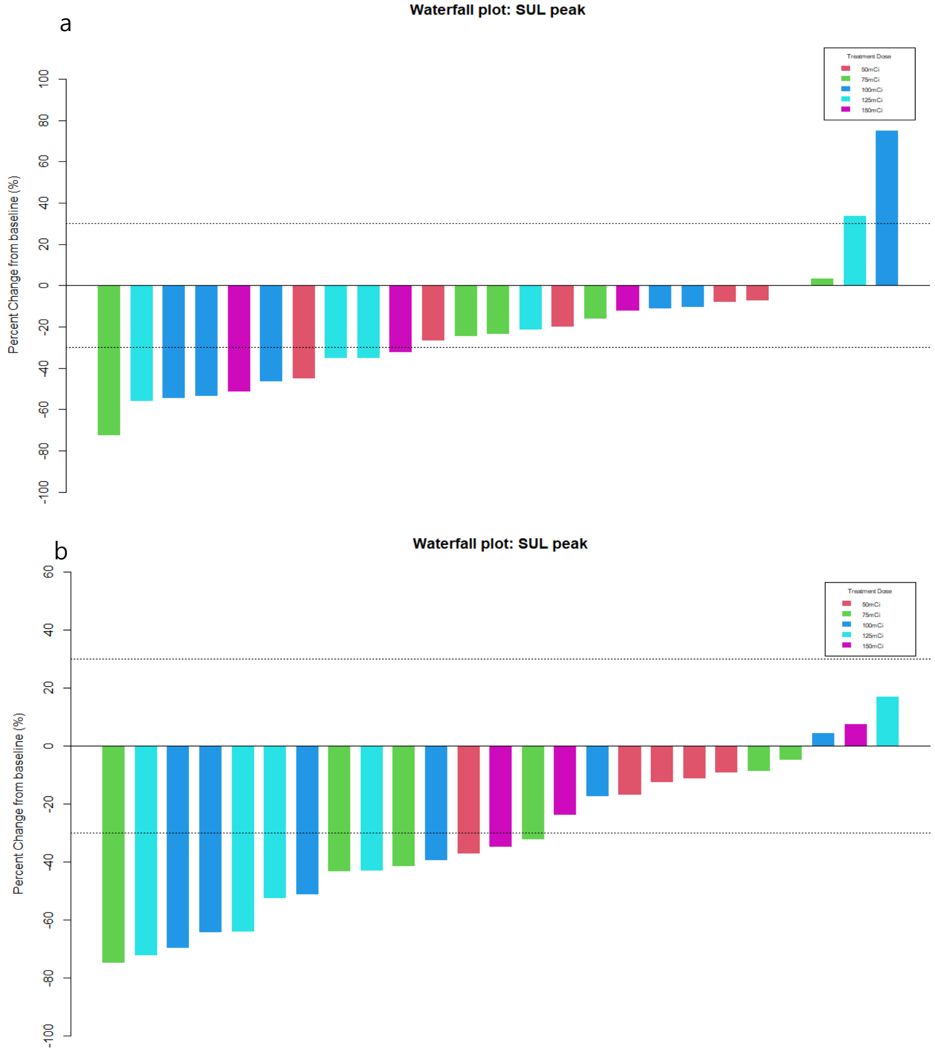

Supplement: Supplementary file 1 [file cancers-14-06225-s001.zip › cancers-2046257-supplementary.pdf]
